# Students’ perspectives on interventions to reduce stress in medical school: A qualitative study

**DOI:** 10.1371/journal.pone.0240587

**Published:** 2020-10-15

**Authors:** Melina Dederichs, Jeannette Weber, Thomas Muth, Peter Angerer, Adrian Loerbroks

**Affiliations:** Institute for Occupational, Social and Environmental Medicine, Centre for Health and Society, Faculty of Medicine, Heinrich-Heine-University of Düsseldorf, Düsseldorf, North Rhine-Westphalia, Germany; University of Birmingham, UNITED KINGDOM

## Abstract

The mental health of medical students remains to be a matter of concern. Numerous setting-based and individual-based interventions for student mental health have been proposed in the literature. However, the student perspective on those interventions has been largely neglected. This study aims to explore how medical students perceive different interventions and if they desire any additional changes with regard to their studies. Eight focus groups with 71 participants were conducted at a large German medical school. Focus groups were recorded, transcribed and content-analyzed using MAXQDA 18. We found that medical students prefer setting-based interventions. Most proposed interventions were on a setting-based level. For instance, students asked for more information on the university’s psychosocial counseling services and for better information management regarding contact persons. Interventions proposed in the literature received mixed reactions: Several participants did not favour a pass/fail grading system. Students considered a peer-to-peer mentoring program for freshmen very helpful. Students had diverse attitudes towards Balint groups. They approved of several self-management courses, most of them being related to time or stress management. Interestingly, the most urgently wanted interventions appear to be rather easy to implement (e.g. a mentoring program). This study explored the medical student perspective on student mental health interventions. Additionally, our study illustrates the benefit and feasibility of involving students early on in the conception of interventions. Further research with a representative sample is needed to obtain broader information on the acceptance of the suggested interventions.

## Introduction

Medical students show a high prevalence of mental illnesses worldwide, such as high levels of depression and depressive symptoms [1].To address medical students’ poor mental health, setting-based and individual-based interventions have been proposed [2–5]. Setting-based interventions aim to improve health by modifying environmental factors, e.g. curricular changes in medical schools [3, 6, 7]. Individual-based approaches, by contrast, seek a change in the individual, e.g., by providing skills that help to cope with stress [8, 9]. Although some interventions have been found to be effective in improving medical students’ wellbeing, there are few consistent findings and the overall quality of data is low [10].

In the following section, we provide an overview of the most important interventions that have been implemented already by some medical schools.

### Interventions described in literature

#### Pass/Fail grading systems

In pass/fail grading systems no mark is given and students do not receive other feedback on their performance than whether they passed or failed (e.g. [11]). A systematic review suggests that pass/fail grading improves medical students’ wellbeing [12] in terms of reduced stress, anxiety or depression and enhanced self-control or satisfaction. Furthermore, pass/fail grading systems seem to improve well-being without negatively affecting academic outcomes [12, 13]. Based on this evidence many medical schools have implemented pass/fail systems already.

#### Peer-to-peer mentoring program

A peer-to-peer mentoring program connects advanced students with freshmen. The latter receive support and insights during their orientation period. Peer-mentoring-programs have been found to reduce stress and to facilitate transition into medical school [14]. However, the quality of data has been described as low. Nevertheless, the notion of peer-to-peer mentoring in medical school has persisted for many years and has been adopted by numerous universities [15, 16].

#### Balint groups

Balint groups are meetings for physicians with a trained facilitator that allow for confidential discussion of challenging patient cases. Traditionally consisting of 6 to 12 physicians, the goal of the groups is to improve the physician-patient-relationship [17, 18]. At the same time Balint groups can help physicians cope with burdensome experiences [19]. Likewise, medical students can be confronted with difficult situations like the sudden death of a patient [20]. Only few studies have investigated the usefulness of Balint groups for medical students. There is inconsistent evidence related to the question whether medical students consider Balint groups helpful or not [21–23].

#### Self-management courses

The term “self-management” includes a range of behavioral interventions and healthful behaviors like emotional management [24]. A systematic review examined 39 studies that evaluated the effectiveness of interventions designed to improve medical students’ wellbeing [8]. Out of 39 studies, 31 focused on individual-based interventions like mindfulness, stress management, psychoeducation, relaxation techniques or yoga. The studies suggested some short-term reduction of anxiety, depression and stress. However, the quality of evidence is low due to weak study designs. Long-term effects could not be determined because they were not assessed in most studies.

### Aim of the present study

The first step in developing suitable interventions to improve medical students’ mental health is to explore which measures medical students find helpful and acceptable themselves. Such user involvement can increase the level of user acceptance [25]. The acceptance of a healthcare intervention is an important prerequisite for its utilization and effectiveness [25, 26]. However, the literature on how students perceive interventions (e.g. in terms of usefulness and acceptance) remains scarce. Most of the interventions that have been tested so far have been devised based on insights into relevant stressors or proposed by experts (e.g. [27]).

Additionally, if students were involved in the process of designing interventions, they often participated as program leaders or in small committees (e.g. the Student Wellness Committee at Vanderbilt School of Medicine [28]). Within the concept of participatory development however, it is important to listen to the full range of opinions to be able to consider all wishes and different perspectives. We therefore conducted a qualitative study with focus groups to gather insights and specific explanations for why students deemed specific interventions useful or not.

Our study aims to answer the following questions:

1. What interventions do medical students themselves suggest and wish for?
2. How do medical students perceive interventions that have been described in the literature (pass/fail grading, a peer-to-peer mentoring program, Balint groups, and self-management courses)?

## Methods

### Participants

Participants were recruited through social media or advertisement on campus of Heinrich-Heine-University of Düsseldorf in Germany. The only criteria for participation were to be currently enrolled as medical student and not having participated at our previous focus groups [29]. Participants of one focus group were recruited through a stress-management seminar. Participation was explicitly on a voluntary basis. All participants gave their informed consent. Students were compensated for their participation with two cinema tickets and one cinema discount card.

Focus groups were conducted until data saturation was reached. Students were grouped into focus groups according to their period of study. This maximised the likelihood that participants could relate to each other in terms of experience regarding classes and exams. Four groups included students in the preclinical part of their studies (year 1 to 3) and four groups included students both from the clinical part (year 4 to 5) as well as students in their clinical internship year (after the second state examination).

### Setting

The focus groups took place in June and July 2019 at the Medical Faculty of Heinrich-Heine-University Düsseldorf. In 2013, the medical faculty introduced a new curriculum. Traditionally, medical students take one final exam organised by the state (state-examination) for each of the three periods of study: the first after the preclinical phase (after year 3), the second after the clinical phase (after year 5) and the last one after one year of practical internship at a hospital. With the new curriculum, the first state examination takes place after the first two years. Permission to conduct this study was given by the ethics committee of the medical faculty of Heinrich-Heine-University of Düsseldorf.

### Material and procedure

After provision of written informed consent, participants were asked to fill out a questionnaire covering demographic data.

The focus groups were conducted by TM, a faculty member and psychologist by training, while MD, a junior researcher and trained psychologist, took notes. The focus groups followed a topic guide developed by MD and TM (S1 File). Both facilitators provide lectures for medical students and are involved in research on medical students’ wellbeing. Participants were informed about the facilitators’ research focus. First, participants were asked about their experience in medical school so far and in which situations they had encountered obstacles or experienced stress. Secondly, students were asked about potential changes and interventions that could contribute to stress reduction in medical school. Previous research at our faculty already pointed out high stress levels and specific stressors of our medical students [29, 30].

To answer the second research question (how the students perceived literature-based interventions), we discussed interventions proposed in the literature with medical students. The topic guide contained a list of the interventions (see introduction section), which were explained by the facilitator and then discussed by the students one after the other. If necessary, the facilitator asked follow-up questions to further explore students’ views. Every single participant was encouraged to contribute to discussions at an early stage.

### Data analysis

All focus groups were audio-recorded, transcribed and content analyzed by MD and JW according to Mayring [31] using MAXQDA 18. JW is experienced with occupational health and qualitative research [29]. Research questions were transformed into deductive main categories (students’ suggestions, interventions from literature). During analysis these categories were further broken down into sub-categories by inductive category formation. After MD completed the first coding round of 25% of the data, AL, an experienced qualitative researcher [29, 30, 32, 33], reviewed the coding scheme. The coding scheme was then adapted and a second coding round was performed by MD. JW coded the data of four groups independently. Both versions were compared and discrepancies resolved. After that, MD recoded the remaining four groups and transferred changes made to the coding system in the previous discussion with JW. Finally, JW and MD discussed the focus groups once more. There was no need for further recoding since there were only minor differences between the analyses of MD and JW. Due to logistic constraints, corrections and feedback on transcripts and research findings were not obtained from study participants.

The completed checklist of consolidated criteria for reporting qualitative research (COREQ; [34]) can be found in S2 File.

## Results

Eight focus groups consisting of 5 to 16 medical students were conducted. Focus groups lasted between 90 and 130 minutes. In total, 71 medical students (56 women) participated. With N = 2816 medical students being enrolled at the medical faculty in the summer semester of 2020, we included 2.5% of the population in our focus groups. Participant age ranged between 19 and 39 years (mean = 23.71; SD = 3.50).

### Generated ideas

All participants engaged in a comprehensive discussion about what they would like to change in medical school to improve student wellbeing. In the following, we will present their proposed starting points for interventions.

#### Regulations for absence of lecturers

Students reported that especially in the clinical study phase, many lecturers did not show up for teaching. Students asked for a substitution or at least a designated contact person.

*“But sometimes I don’t know*. *Okay*, *if nobody shows up [for the lecture]*, *whom do I ask*? *And then I contact the ward nurse*. *And she calls the medical referee and he then calls someone else*. *Somehow it would be nice if there were a list or something where you knew*, *okay*, *nobody is showing up for the lecture*, *so I can call this number*.”

#### Attendance

Attendance rules were perceived to be too strict. According to the study regulations, an attendance of at least 85% presence per subject is required. However, if one subject consists of three appointments for example, all three seminars have to be attended. By attending only two of them, the presence would be 67%, thus below 85%. Students therefore requested that if one misses class due to sickness or death of a family member, an exception should be granted.

*“I think the pressure that is put on us is relatively high*. *[Regarding] absence*, *it is obvious that there is a need to keep it within a limit*. *But I think that if someone really wants to attend a funeral or has a sick leave certificate*, *then not attending should be permissible*. *It is unacceptable that we have to attend our seminars sick because otherwise we would have to repeat the semester […]*. *A fellow student of mine tried to stand next to the dissection table with a 40-degrees fever*, *just trying to keep her eyes open*. *She didn’t pass the exam because she didn’t have time to recover*.”

According to the participating students, there should also be the option to complete a compensatory task, for instance, holding a presentation on the subject that was missed. As another solution for not missing a mandatory event, participants suggested switching groups with their peers for the required event only. Due to the high number of mandatory classes during the preclinical phase, students wished for less mandatory attendance during this phase.

#### Coordination of practical and theoretical phases

The Düsseldorf medical curriculum combines phases of theoretical studying (lectures) and practical experience (clinical traineeship). Students wished that the curriculum is adapted so that theoretical and practical lectures as well as corresponding tutorials are better aligned and deal with the same subjects. This was believed to increase their learning effect.

*“I think it would carry our studies forward in terms of quality if practical phases matched theoretical lessons*, *so that you get the opportunity to deal with one topic over a period of eight weeks*, *to repeat things*. *And then this would allow for things to consolidate and settle*.”

Students noted that the workload between theoretical and practical phases fluctuates significantly with high workload during theoretical phases and low workload during practical phases. Therefore, they wished for shorter practical phases to distribute the workload more evenly.

Often, the lecture-free week takes place after the clinical traineeship. However, students would rather have a week off for studying after completing the theoretical lectures when they find to have more content to repeat and memorize.

#### Counseling

Participants pointed out that they would benefit from a broader psychological support system. Regarding the on-campus counseling service, students wished for longer opening hours as well as opening hours that are compatible with their schedules. Free of cost confidential counseling services were perceived as useful for reaching out to students to address mental health issues. Overall, students wished for more information regarding mental health problems and interventions.

*“[…] especially at the beginning of our studies there is a need for more lectures in our curriculum that address this topic*. *Like how do I deal with stressful situations*? *Whom can I ask for help*? *So that it becomes more normal and isn’t only [mentioned] at the funeral of a fellow student who put an end to his life*. *[…] Things like that [informational event] should be part of the curriculum*. *That is how they are established*.”

Possible solutions students brought up were for instance a mandatory lecture about stress related to medical school, coping strategies and support contacts. Additionally, students suggested that a newsletter should be emailed close to the first state examination to present information on emergency contacts and counseling services.

#### Registration processes for elective subjects

Students explained that when they want to register for a subject, they have to log in to an online portal. There they would have to wait until midnight until the registration process opens. They further explained that registrations are allocated on a first come, first serve basis. Therefore, students stated that without access to a fast and reliable internet connection at that time they would not be able to take the class they prefer.

Students would like this procedure be replaced by a selection based on preference. In this procedure, students could indicate their preference within a certain period. Then they would be matched according to their choice. If there is no personnel to do this task, some students suggested that at least the first come-first serve procedure was opened during daytime.

#### Information management

Students reported a lack of information management in medical school. They requested a comprehensive online portal containing information on classes and exams. Moreover, students expressed that they would benefit from an introductory lecture for each new topic block that contains all relevant administrative information, e.g. on how to register for an exam. A FAQ that addresses most urgent questions concerning the first state examination was considered useful.

*“The search for information is extremely tiring*. *If you are already stressed out and are searching for specific information but just can’t find it*, *it’s incredibly frustrating*. *“[There should be] a neatly organized guide [about the first state examination] or some information including frequently asked questions and answers […]*”

#### Online lectures

Many students raised the topic of online lectures. They perceived online lectures as an efficient measure to reduce stress and improve flexibility. Especially commuters were thought to profit significantly from it. Online lectures were thought to be a great learning tool and aid with the preparation of future seminars. One student requested a contact person in case any questions arise during a session.

*“I think it would be great to have this opportunity*. *I personally learn at night*. *In the morning*, *I don’t pick up much*, *but at night I am really diligent*. *And it were a lot easier to combine family*, *job and university*. *That would be a huge advantage*.”

#### Refresher courses

Students stated that they would benefit from refresher courses before the second state examination.

*“I believe it would be pretty fair*, *for instance*, *to have courses that prepare you for the state examination in practical terms during the semester*. *That would be*, *I think*, *not bad*.”

#### Examinations

Students reported that their exams cover several subjects at once. Students asked for a restricted number of subjects within the same exam. They reported that this could be achieved if exams included more questions on the same subject per exam instead of the same subject posing only a few questions per exam over several exams.

*„Now for instance [in this exam] there were like 15 different subjects*. *Often with only a few questions*, *but I lost track*. *I forgot to learn five of them because the list [of subjects] was so long*. *I would prefer it to be limited*. *Ten subjects*, *that you can keep in mind and study*.”

They also suggested grades acquired in seminars should be added to the exam score and act as a buffer.

It was discussed whether more open-ended questions instead of multiple-choice questions would be beneficial. Some students stated that they could explain their knowledge better in open-ended questions. Moreover, it would motivate them to learn more details. Others preferred the existing multiple-choice format.

#### Teaching content

Participants made a range of suggestions to improve the quality of teaching.

For instance, students wished for a contact person to report verbal harassment of students like depreciative comments by teaching staff. Exact teaching guidelines containing learning goals for every subject were considered potentially helpful.

*“I think it is unacceptable that people are treated unfairly*. *That some receive a very good exam preparation and others a very bad preparation*, *not learning anything in the course*. *I think lecturers should be encouraged to get some sort of catalogue on what they should explain and who needs to be taught what […]*”

They also wished for more covering of content relevant for the second state examination. A perceived lack of interest and skill of teaching staff was thought to be solved through employing senior students or external referents. They asked for more interactive and clinically orientated teaching.

#### Elective subjects

In total, 14 elective subjects have to be completed over the course of five years at the Medical School in Düsseldorf. Some of the subjects are graded. Participants raised the idea to reduce the total number of required courses to reduce stress and provide more time for state exam preparation.

*“It is good that we have elective subjects*. *But there are too many*. *And it is stressful*, *to have eight elective subjects here and another six elective subjects there*. *It would be great if we had less*.”

Alternatively, additional credit for scientific projects, clinical lectures or extracurricular activities was suggested.

#### Clinical traineeship

Students asked to move the clinical traineeships towards the end of the medical curriculum, because students felt that they are often confronted with medical conditions for which they are not prepared yet and often clinicians presuppose content they have not dealt with yet.

*“In the last general physician traineeship*, *I benefitted because I knew something*. *I knew the medications and I felt like I could say something about every [patient]*. *And there I really learned something and could pick [new] things up*. *But in the general physician traineeship before that … It’s just too early*, *in my opinion it doesn’t yield a learning effect*, *it might as well be dropped*.”

They also suggested informing clinicians about what the students have learned so far in order for them to have a more realistic estimate of what a student can be requested to do and what exceeds their knowledge.

Students reported frequent difficulties in their clinical traineeships. For instance, clinicians that are supposed to teach them are often not available and, if they are present, students felt that they do not take time to teach them adequately. More available contact persons or a central complaints office that assists them were mentioned as a possible solution to this issue.

#### Preparation for the second state examination

Students wished for lecture free and exam free time for preparation before the second state examination. They requested to have at least 100 days to dedicate completely to studying. This way they stated they could follow the learning schedule they wanted to use (so called “100-day study plan” which is well-known and widely used by medical students in Germany).

*“It is a question of planning*. *Then*, *if you have these one hundred days*, *I think that would help a lot*. *And it would be nice if it wasn’t exactly one hundred days but a couple of days more because maybe you don’t study seven days a week*.”

Participants had many ideas on how to provide more time to study. Participants proposed shortening clinical trainings, or to move them towards the end of the medical curriculum to avoid that students have to pass other exams right before the second state examination. Students were willing to complete their clinical training during semester break or to begin the semester early to provide more preparation time for the second state examination.

*“I think everyone would be willing to sacrifice a little*, *like one week of recreational time during this period to have a longer preparation time for the second state examination*.”

*“If all semester breaks were shortened by one week*, *I think that would still provide enough free time*. *Then*, *at the end [of medical school]*, *we would have another eight weeks [to study]*.”

### Measures proposed in the literature

#### Pass-fail system

There was no consensus among the students in our focus groups related to the usefulness of pass-fail systems. Some were in favour of eliminating grades because it would reduce pressure and stop demotivation through bad grades. Others in favour of the pass-fail system argued grades did not have any informative value regarding their ability as a physician anyway and were thus of no importance. Students opposing the pass-fail system reported to appreciate the incentive they received from good grades and to appreciate the feedback to better understand whether they had learned enough.

*“To be honest*, *I use my performance for orientation*. *It helps me to see what I did wrong […]*. *Or it gives me an incentive to do better*. *[…]*”

Students depending on a scholarship or on receiving particularly good grades raised concerns regarding their ability to compete and prove their performance. Scholarship holders among the students reported that in order to keep their scholarship, they had to prove to belong to the top students in their semester. Without grades, this might be difficult to prove and students from other universities might have an advantage. In Düsseldorf, the final grade for the first state examination is calculated as a cumulative sum score of grades from the first to the third year and of the performance on the day of the state examination. Students argued that this way they did not solely depend on one’s day performance and therefore the stress on the day of the first state examination was reduced. Some preferred keeping grades to maintain this system. However, students suggested alterations of the pass-fail system. They were in favour of eliminating grades from some subjects to reduce stress but maintain the current system of collecting grades for the first state examination.

#### Mentoring

Almost all participants were in favour of a mentoring program. They appreciated it for its easy access and voluntary nature. The idea of having a permanent contact person that provides not only informational and emotional support but also further networking opportunities to higher semesters was welcomed. The personal experience and organizational knowledge of a senior student was thought to reduce stress significantly.

*“I would have liked having someone who told me ‘Everything is fine*, *this is normal*. *I experienced it too*.*’ Especially at the beginning of medical school… I had just moved here… and everything was new*. *Having someone who can answer questions related to studying or simply help you*.”

Many students in senior semesters expressed the desire to become a mentor themselves. Those who did not want a mentoring program stated they already established such a contact to a senior student by themselves. There were many suggestions regarding the design of a mentoring program. A pool with volunteers, possibly on an online-based platform was proposed. Students further proposed that mentors should receive some form of compensation, either monetary or in a non-monetary form, like an honorary position. Students argued that their willingness to participate in a mentoring program also depended on the mentor’s personality. They feared that overly performance-driven students might be a bad role model in terms of stress management.

*“It truly depends on who is doing [the mentoring]*. *If it is some extreme medical student*, *who learns at night and*, *I don’t know*, *whenever possible*, *on the loo*, *whenever*, *then I would politely decline*.”

#### Balint groups

The idea of participating in a Balint group received mixed feedback. Those in favour of Balint groups requested that it should be on a voluntary basis and regularly starting with the beginning of medical school. Especially for difficult patient cases, it was perceived as potentially helpful.

*“Having the possibility to talk to others can reduce fears […]*. *And you learn how to handle [your fears]*.””

Students who would not want to participate in a Balint group stated that they preferred being mentored by their peers or supervising physicians. They reported to rely on their own social networks for emotional support. Some students expressed fear of stigmatisation due to participation in a Balint group. They worried about talking freely about unpleasant experiences and being stigmatized by their peers. Therefore, they would not want to participate.

#### Self-management courses

Students were interested in learning self-management strategies or competences to strengthen their resilience. In this context, they wished for subjects on stress-management and relaxation techniques.

*“It is nice that we know what to do with our patients when they feel bad*. *But it is bad that we don’t have any guidelines ready at hand on what to do if we ourselves feel bad*.”

Moreover, students wished for a time-management course and more capacities in an existing elective subject deals with mind-body-medicine. Participants stated they would benefit from courses that covered efficient learning strategies and presentation skills. There was little consensus among participants regarding the question when and how those classes should be implemented in their curriculum. In general, students perceived lack of time as a barrier to participate in a non-obligatory class. Some students suggested that it should be an ongoing offer over a long period of time where students could decide for themselves every week whether they wanted to participate or not. Students preferred to schedule such courses at the beginning of medical school and shortly before the first state examination.

General dissatisfaction with already existing self-management courses on campus was expressed. Students either perceived lack of information about those classes, lack of quality of available classes or limited capacity to enroll all interested students.

## Discussion

The aim of the present study was to explore which interventions students suggest to improve their mental health and to discuss interventions suggested in the literature. In our eight focus groups, students suggested specific solutions to their perceived obstacles in medical school. Interventions that students proposed most frequently pertained to setting-based aspects such as curricular changes, improved information management and new regulations concerning absence of teaching staff and students.

Many of the suggested interventions, such as online lectures, appear rather easy to implement. Due to the current COVID-19 pandemic (time of publication; [35]), our faculty addressed the wish for more online lectures by creating new digital learning opportunities and structures. Eventually these structures will persist after the university switches to conventional lectures once again. It is also striking that many of the suggested interventions might not only reduce stress, but also improve several aspects of teaching (e.g. the coordination of practical and theoretical phases). This suggests that improvement of well-being and improvement of academic outcomes go into the same direction. In contrast to students’ wish for setting-based interventions, most interventions being proposed in the literature focus on the individual [8]. However, such interventions are believed to not tackle the root of the problem [36]. It is argued that instead of teaching students how to cope with stress, the causes for stress need to be addressed [36]. Some individual-based interventions (e.g. stress-management courses) might even have the opposite effect by being an addition to existing classes and workload. It is further of interest that we observed that a significant number of our participants did *not* favor the interventions suggested by the literature. For instance, several students did not prefer a switch to a pass/fail grading system. These students perceived grades as helpful, were concerned that the absence of grades would have a negative impact on their academic performance, or would cause increased stress at the first state examination. However, this latter concern is not supported by the data [12, 37].

It is possible that the idea of a pass/fail grading system elicits discomfort among our students because it is rather uncommon in Germany. When the pass/fail system was first introduced in the US similar concerns were raised [38]. A shift to a pass/fail system was perceived as a disadvantage for students and students coming from a university with pass/fail grading were thought to experience difficulties finding a residency placement. Therefore, the authors did not recommend such a transition [38]. However, a recent study showed that a pass/fail grading does not curtail career prospects [29]. Specifically, there were no differences in residency placement (receiving a placement at all and percentage of students that receive top specialty choices) and overall academic performance between pass/fail and tiered grading system cohorts [29].

Besides pass/fail grading being uncommon in Germany, it would also require a change of the persisting score calculation for the first state examination. Additionally, it would require a cultural shift towards a non-competitive environment among all students in order to be successful. Therefore, instead of fully switching to pass/fail grading only, a mixed approach might be more feasible in which pass/fail grading could be adopted in some subjects while other subject are still graded. In this approach, grades still contribute to the result of the state examination. This would address medical students’ preference to accumulate marks and thereby reduce stress during the first state examination without a prompt change that requires time for adaptation.

Possible mentoring programs by senior students were well received by the participants in our study. More advanced students were believed to provide invaluable informational and emotional support. Since such a program has relatively low costs and was also favored by potential mentors, we propose starting a peer-to-peer mentoring program at our university as recommended by others [39].

Balint groups received mixed reactions. This is in line with previous research suggesting indifferent to mildly positive student attitudes towards Balint groups [21, 22]. A qualitative study from Finland reported a more positive evaluation of Balint groups [23]. Here, students reported being satisfied with their participation and benefiting from the groups [23]. In our present study, especially those students who had already encountered a difficult situation with a patient stated that they would be grateful for this kind of opportunity and support. Therefore, the implementation of a Balint group with voluntary participation on request as suggested by students could be considered. It could build on already established peer-support-programs such as the programs at Brigham and Women’s Hospital in Boston, which has repeatedly served as a model for peer-support-programs [40].

Students themselves requested a variety of self-management courses. Most were interested in learning relaxation techniques and how to deal with stress. They stated that existing offers, like a time management class, are less attractive because their study curriculum does not allow for the implementation of taught strategies in this class. Overall, they expressed the wish that classes to improve resilience should be accessible to everyone. Some stress management courses (e.g. “stress management” or “mind-body-medicine”) are only offered as elective subjects and are therefore only available for a fraction of the students. We suggest that all students receive a basic stress-management training and psychoeducation, preferably at the beginning of the curriculum. At some universities, such as the Monash University medical school in Australia, mindfulness and stress management have been part of the curriculum for many years [41]. Since some studies found reliable short-term effects [8, 42], students could benefit from it in most stressful times without the concern of losing time to study. For the latter reason we also suggest that more classes that address mental health are offered. In addition to face-to-face training, e-mental-health-solutions could be made accessible for students for quick support. Students’ wish for e-learning opportunities suggests that they are open to digital formats that grant them more independence in terms of time and location of use. Furthermore, information on specific counseling opportunities should be made more accessible. It is possible that raising awareness of medical students’ mental health will reduce fear of stigmatization by peers which was mentioned as a concern in the context of Balint groups.

Overall, we find that students proposed more setting-based interventions than individual-based interventions. This is in sharp contrast with the persistent emphasis on individual-based interventions in medical schools [8–10, 43]. Importantly, while setting-based interventions are sometimes considered expensive or difficult to implement [7], most ideas in the focus groups (e.g. attendance rules, a new course selection procedure, teaching guidelines) seem easily feasible and resource-friendly and will not only improve wellbeing, but also academic performance.

### Strengths and limitations

A strength of this study is its rich data, which were collected among as much as 71 students from a broad range of semesters in eight focus groups until data saturation was reached.

However, the generalizability of our findings may be limited since only students from the medical school at the University of Düsseldorf were included in this study. Our findings might be specific to issues and obstacles encountered at universities in Germany and therefore only somewhat transferable to universities in other countries.

When we asked the participants about their opinions on a pass/fail grading system, we did not clarify in detail whether they would still like feedback on their performance if a pass/fail grading system was implemented. Thus, we do not know to what extent students perceive a pass/fail grading system and feedback on their performances mutually exclusive.

Further, we cannot rule out selection bias. One might argue that only those students suffering from stress or those who are especially dissatisfied with medical school attended the focus groups. On the other hand, one might assume that those students experiencing a high level of stress choose not to participate because of the additional workload. The fact that focus group facilitators were members of the teaching staff and that one focus group was held within a class on stress management could also have affected the observations. MD’s and TM’s position as a teaching staff member might have elicited a social desirability bias and reduced willingness to share sensitive topics. However, also in this seminar, we felt that students spoke very openly about their issues. The contents of this focus group did not differ thematically from the other focus groups.

This study explored which type of interventions students consider acceptable and useful. Based on our data we are however unable to evaluate the feasibility and effectiveness of proposed interventions. However, we believe that only interventions that are favored by students and address their specific needs will be successful.

## Conclusions

In contrast to interventions to improve medical students’ mental health that are proposed in the literature, we find that medical students mostly proposed interventions on a setting level rather than on an individual level. Importantly, many interventions suggested by the students are low-cost and easy to implement. We believe that considering the student perspective is a key factor in designing mental health interventions. Further research with a representative sample is needed to obtain more generalizable information on the acceptance of the proposed interventions and to test them in terms of feasibility and effectiveness by using both qualitative and quantitative approaches.

## Supporting information

S1 File Focus group guide. (PDF)

S2 File Completed checklist of the consolidated criteria for reporting qualitative research (COREQ). (PDF)
