# Supplementary material for: Students’ perspectives on interventions to reduce stress in medical school: A qualitative study
Source: PLoS One. 2020 Oct 15;15(10):e0240587. doi: 10.1371/journal.pone.0240587 (PMC7561099; doi:10.1371/journal.pone.0240587)
Supplement: S1 File — (PDF) [file pone.0240587.s001.pdf]

Supporting Information File 1

Focus group guide according to final research questions

Note to facilitator: Enable everyone to speak, but not necessarily in a fixed order. Ensure that discussions do not wander off to stressors only or the evaluation of specific courses.

1) Introductory question:

   "In your medical studies, what was easy for you, where did you encounter problems? Which are or were for you the main burdens in your studies?"

2) Key question regarding ideas for interventions:

   "Which changes or interventions could help you reduce stress in medical school?"

3) Key question regarding changes from the literature:

   "In the following we want to present you a number of interventions that have been proposed in the literature. What do you think of –"

   a) A pass/fail grading system?

   b) A peer-to-peer mentoring program?

   c) Balint-groups?

   d) Self-management courses (e.g. time- or stress-management, mindfulness)?

4) Outlook:

   "Is there anything else you would like to add? Have we discussed everything that is important for you? "

   "Thank you."
