# Supplementary material for: Students’ perspectives on interventions to reduce stress in medical school: A qualitative study
Source: PLoS One. 2020 Oct 15;15(10):e0240587. doi: 10.1371/journal.pone.0240587 (PMC7561099; doi:10.1371/journal.pone.0240587)
Supplement: S2 File — (PDF) [file pone.0240587.s002.pdf]

Supporting Information File 2

Completed checklist of the consolidated criteria for reporting qualitative research (COREQ; (1))

No	Item	Description
**Domain 1: Research team and reflexivity**		
Personal characteristics		
1.	Facilitator	Thomas Muth (MD took notes)
2.	Credentials	Melina Dederichs (MSc), Jeannette Weber (MPH), Dr. Thomas Muth, Prof. Dr. Peter Angerer, PD Dr. Adrian Loerbroks
3.	Occupation	MD, JW: research associate; TM, AL: senior research associate; PA: university professor
4.	Gender	MD, JW: female; TM, PA, AL: male
5.	Experience and training	MD: educational background in psychology, practical experience in occupational health research, qualitative research and teaching; JW: educational background in biomedical science and public health, practical experience in occupational health research; SS: medical student; TM: educational background in psychology and public health, experience in occupational health research, qualitative research and teaching; PA: educational background in human medical studies, experience as a clinical doctor and in occupational health research; AL: educational background in epidemiology and health sciences, experience in occupational health research, qualitative research and teaching
Relationship with participants		
6.	Relationship established	No
7.	Participant knowledge of facilitator	Participants knew that TM and MD were working as a research associate on student health
8.	Facilitator characteristics	No other characteristics were reported about the facilitator
**Domain 2: Study design**		
Theoretical framework		
9.	Methodological orientation and theory	Qualitative content analysis by Mayring
Participant selection		
10.	Sampling	Convenience sampling
11.	Method of approach	Via advertisement on campus, social media or personal contact
12.	Sample size	71 participants
13.	Non-participation	Not applicable
Setting		
14.	Setting of data collection	Conference room at university
15.	Presence of non-participants	MD took field notes
16.	Description of sample	$Mean_{age}$=23,71 (range: 19-39 years); female = 79%, male = 21%
Data collection		
17.	Interview guide	Provided as supplemental material
18.	Repeat interviews	None
19.	Audio/visual recording	Audio recording
20.	Field notes	Yes
21.	Duration	Circa 90 – 130 minutes
22.	Data saturation	Yes
23.	Transcripts returned	No
**Domain 3: Analysis and findings**		
24.	Number of data coders	Three
25.	Description of coding tree	Yes (in the result section pp. 6-16)
26.	Derivation of themes	Deductive coding: suggestions for improvement, measures from literature; all other categories were inductively coded
27.	Software	MaxQDA 18
28.	Participant checking	No
Reporting		
29.	Quotations presented	Yes
30.	Data and findings consistent	Yes
31.	Clarity of major themes	Yes
32.	Clarity of minor themes	Yes

1. Tong A, Sainsbury P, Craig J. Consolidated criteria for reporting qualitative research (COREQ): a 32-item checklist for interviews and focus groups. International Journal for Quality in Health Care. 2007;19(6):349-357.
